# Few-Shot Object Detection Using Multimodal Sensor Systems of Unmanned Surface Vehicles

**DOI:** 10.3390/s22041511

**Published:** 2022-02-15

**Authors:** Bowei Hong, Yuandong Zhou, Huacheng Qin, Zhiqiang Wei, Hao Liu, Yongquan Yang

**Affiliations:** College of Computer Science and Technology, Ocean University of China, Qingdao 266100, China; hongbowei@ouc.edu.cn (B.H.); zhouyuandong@stu.ouc.edu.cn (Y.Z.); qinhuacheng@stu.ouc.edu.cn (H.Q.); weizhiqiang@ouc.edu.cn (Z.W.); liu.hao@ouc.edu.cn (H.L.)

**Keywords:** unmanned surface vehicle, object detection, few-shot learning

## Abstract

The object detection algorithm is a key component for the autonomous operation of unmanned surface vehicles (USVs). However, owing to complex marine conditions, it is difficult to obtain large-scale, fully labeled surface object datasets. Shipborne sensors are often susceptible to external interference and have unsatisfying performance, compromising the results of traditional object detection tasks. In this paper, a few-shot surface object detection method is proposed based on multimodal sensor systems for USVs. The multi-modal sensors were used for three-dimensional object detection, and the ability of USVs to detect moving objects was enhanced, realizing metric learning-based few-shot object detection for USVs. Compared with conventional methods, the proposed method enhanced the classification results of few-shot tasks. The proposed approach achieves relatively better performance in three sampled sets of well-known datasets, i.e., 2%, 10%, 5% on average precision (AP) and 28%, 24%, 24% on average orientation similarity (AOS). Therefore, this study can be potentially used for various applications where the number of labeled data is not enough to acquire a compromising result.

## 1. Introduction

In recent years, with growing global interest in commercial, scientific, and military issues associated with the marine environment, there has been a corresponding growth in demand for the development of unmanned surface vehicles (USVs) with advanced guidance, navigation, and control (GNC) capabilities [1]. Object detection plays an important role in the marine navigation system of an USV and its dynamic environment perception [2,3,4,5]. However, it is also one of the difficulties facing USV navigation. The unique conditions experienced in the marine environment, such as winds, waves, sea fog, water reflection, another vehicle crossing through and so on, make it difficult to apply conventional object detection methods directly. The method has to be modified to adjust those situations above since the complex marine situations could impact the performance of classical object detection [2,6].

Light detection and ranging LiDAR systems and visual sensors are the main shipborne sensors to be extensively adopted in the environment perception system of modern USVs. LiDAR is good at near-range obstacle detection, with high depth resolution and accuracy, while a camera is simple and low-weight in practical applications, with high lateral and temporal resolution. However, the performance of LiDAR is limited by sensor noise, calibration errors, sensitivity to the environment and USV motion. Meanwhile, low depth resolution [7], poor accuracy, and a lack of real-time detection make the camera unable to meet the demand of marine environment perception [8,9], too. As single sensor-based object detection methods do not perform well in surface object detection [10], we expect that the fusion of point cloud and image data can do better. Reference [11] showed that the fusion of LiDAR point cloud and image data achieved higher performance, which supports our assumption.

Another problem in the recognition of surrounding obstacles is the lack of a training dataset. Collecting and labeling data is typically expensive, so researchers perform experiments on a public dataset to prove the validity of their models. However, for a complex marine environment, there are no such large-scale public datasets yet. Thus, we proposed a few-shot object detection method, which can learn a class from a few labeled examples without missing small-scale target objects.

**Figure 1:**
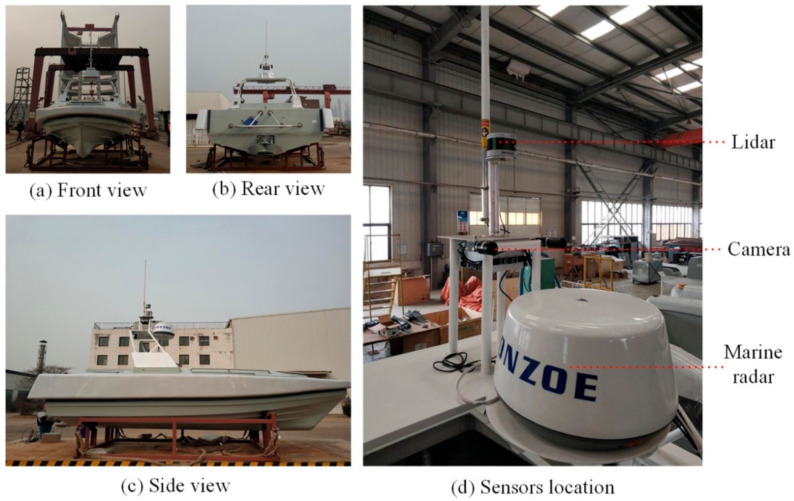
Multimodal sensor system for USVs.

The USV and its airborne multimodal sensor system we used in this paper is shown in Figure 1. The multimodal sensor system is composed of LiDAR, a high-resolution industrial camera, maritime radar, and inertial navigation equipment. LiDAR and the camera are used for surface object detection, the maritime radar is used to detect, locate, and avoid long-distance obstacles, and the inertial navigation equipment is used to position the USV accurately and adjust the course angle.

**Figure 2:**
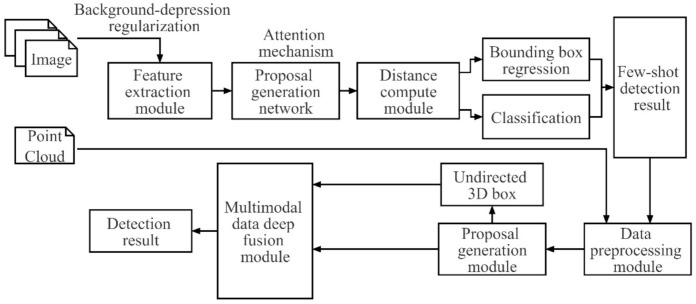
Prototype model of the few-shot object detection based on multimodal sensors.

In this paper, we propose a few-shot surface object detection model that takes multimodal data as input and predicts the full 3D extent of objects on the water surface. The proposed network is supposed to detect obstacles and recognize targets (location and position) on a small-scale dataset. As illustrated in Figure 2, the few-shot surface object detection model consists of two parts: a few-shot object detection network and a 3D object detection network. The few-shot object detection network utilizes small-scale datasets (image) to perform the detection of objects, which consists of five components: feature extraction module, proposal generation network, distance compute module, bounding box regression and classification. Then, detection results (labelled image and point cloud) are screened to extract categories of key surface objects to locate the ship objects that will be taken as target objects for 3D object detection.

The proposed model makes the following contributions:

1. Inspired by ResNet50 [12], we introduce a regularization term for background suppression into the feature extractor, which enhances the extraction of foreground object regions and improves the accuracy of the region proposal box;
2. We propose a feature fusion region proposal network (RPN) that utilizes multiple modalities to produce region proposals for small classes;
3. We propose a few-shot learning module based on metric learning, with a better label classification result and a more accurate localization;
4. We propose a key object detection method on the water surface that utilizes multimodal data as the data source. Our approach outperformed the state-of-the-art approach by around 2%, 10%, 5% on AP and 28%, 24%, 24% on AOS in three sampled sets of well-known datasets;
5. This study can be potentially used for various applications where the number of labeled data is not enough to acquire a compromising result.

## 2. Related Work

Few-shot object detection is a fusion of conventional object detection and few-shot learning, which aims to obtain a model with good generalization capability on small-scale samples. Since Girshick [13] introduced deep convolutional networks to object detection in 2014, modern object detection has seen tremendous progress. As one of the representative architectures, region-based convolutional neural network (R-CNN) has generally been explored in the context of few-shot object detection [14,15,16,17,18,19,20], taking Fast R-CNN as the backbone network for object detection. Some research has been based on one-stage object detection algorithms, such as you-only-look-once (YOLO) [9] and single-shot detection (SSD) [8]. Kang [21] and Deng [22] took YOLOv2 [23] and YOLOv3 [24] as the backbone network, respectively, while Yang [25] took SSD as the backbone network. The challenge of few-shot object detection is not only to identify targets accurately but also to determine the exact position and orientation of targets.

Table 1 indicates the characteristics of the main few-shot object detection methods among which metric-learning-based methods have solved the few-shot classification and few-shot object detection problems [26,27]. The metric learning method has been further improved by the RepMet network [28]. Specifically, RepMet replaced the conventional R-CNN classifier head with a distance metric learning (DML) subnet and keeps the feature pyramid network-deformable convolutional network (FPN-DCN) [29] as the backbone network, which has achieved promising detection results. Our few-shot object detection model was designed based on R-CNN, and RepMet was used as the baseline.

Gupta et al. [30] proposed adding depth information to the 2D object detection framework R-CNN. Specifically, by learning Depth Map features using Depth CNN and 2D image features using RGB CNN and SVM classification, 3D object detection with depth information was achieved. The 3D region proposal network (RPN) [31] was later created based on the Faster R-CNN framework, which takes the 3D volume scene in RGB-D images as the input and outputs 3D target bounding boxes. It is able to detect obstructed objects. However, as the 3D RPN is based on the 2D object detection framework, and there are limitations to the physical properties of the camera, there are problems such as poor detection results and decreased detection accuracy as a result of the information loss of small objects during the 2D–3D conversion.

In a study by Chen et al. [11], the MV3D object detection framework was proposed, which extends image-based RPN to 3D by corresponding every pixel in the BEV feature map to multiple prior 3D anchors [32]. The front view and the BEV of laser point cloud data are used to represent the object position information, which is then integrated with the image information to predict the directed 3D bounding box. However, MV3D is unable to achieve real-time processing, especially in detecting small objects, and cannot adapt to multi-object scenes in the vertical direction well. AVOD [32], an improvement of MV3D, simplifies the input of MV3D while ensuring the accuracy of the 3D position information of the object. The pre-processing and calculation are more efficient, as only the BEV of the point cloud and image data is used. Cheng et al. [33] proposed a radar-vision fusion-based method that can be applied to small object detection for USVs. However, the study focused on small object detection (floating bottle detection) without concerning 3D information, e.g., the orientation of objects, which is essential for moving object detection. In previous research [34], we reduced the computation requirements with a high accuracy of 3D object detection on the sea surface, which meets the demands of marine environment perception for USVs. In this paper, we make further improvement on the accuracy of 3D detection and the reduction of computational requirements at a realistic level. Instead of performing detection at first sight, the proposed 3D detection network picks up target objects according to the classification result of proposed few-shot object detection model first, then it performs accurate oriented 3D bounding box regression and classification to predict the extent, and the orientation of both static objects and moving objects on the water surface.

## 3. Few-Shot Object Detection Network Based on Metric Learning

**Figure 3:**
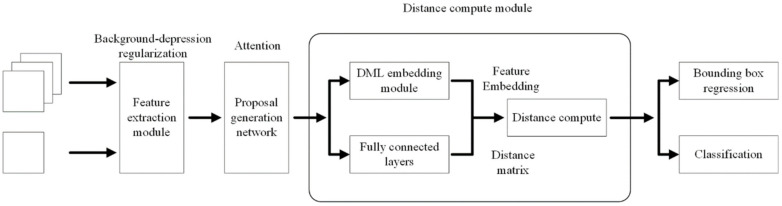
Network structure of the few-shot surface object detection method based on metric learning.

As shown in Figure 3, the first part of the proposed few-shot object detection network based on metric learning consists of three modules: feature extraction module, enhanced region proposal module, and distance compute module.

### 3.1. Feature Extraction Module

The feature extraction module uses modified ResNet50 [12] as the feature extraction network. To eliminate the interference of the complex background on foreground objects in the few-shot scenes, a regularization term for background suppression was introduced to optimize the training of the feature extraction network, as shown in Equation (1). In background suppression regularization, the basic features of the training samples of target domain dataset are first extracted. Second, the labeled bounding box is projected to the basic features to obtain the background feature region. Lastly, the L2 paradigm computing result of the background feature region is taken as the auxiliary loss item of model training. Through background suppression, the extraction of foreground objects region can be enhanced, and the accuracy of the region proposal box can be improved, which is of great significance to the training of the few-shot object detection model. The regularization term is calculated as follows:

(1) $$L_{BD}=\|F_{BD}{\|}_{2}.$$

### 3.2. Proposal Generation Network

**Figure 4:**
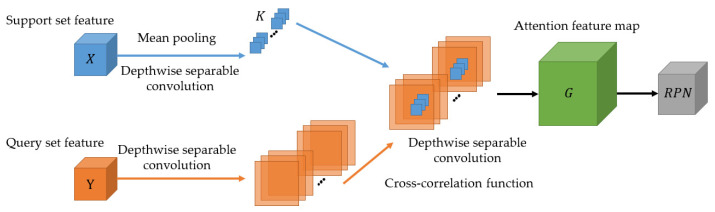
Generation of the attention feature map.

To enhance the filtering results of RPN on the background-category candidate box and the other non-support category candidate box, the enhanced region proposal network inputs the information of the support set into the RPN using the attention mechanism. Specifically, the features are extracted for the support set and query set, and the eigenvectors are noted as *X* and *Y*, respectively. Then, mean pooling [35] is carried out for *X*, the convolution operation is carried for *Y* to extract the attention feature map *G*, and the convolution operation of *G* is performed to generate the candidate box. Lastly, the classification and regression branches are input through the ROI Align module [36]. The generation of the attention feature map *G* is shown in Equation (2):

(2) $$G_{h,w,c}=\sum_{i,j}X_{i,j,c}Y_{h+i-1,w+j-1,c},\;i,i\in \left\{1,\ldots ,S\right\},$$

where *h*, *w*, and *c* are the height, width, and number of channels of the feature map, respectively. *X* is the features of the support set, and $X\in t^{S\times S\times C}$, *Y* is the features of the query set, and $Y\in t^{H\times W\times C}$. *H* and *W* are the height and width value, respectively, of the feature map *Y*. The enhanced region proposal network learns the similarities in the features of the support set and the query set through depth-wise separable convolution and then generates the candidate box based on the similarities, as shown in Figure 4.

### 3.3. Few-Shot Learning Module Based on Metric Learning

After ROI Align pooling of the candidate box generated by the enhanced region proposal modules, the eigenvector *M* is obtained, which is sent to the classification branch and the regression branch of the metric-based few-shot learning module. After that, the label classification result and location information of the bounding box are obtained. In the classification branch, the representation vector of the eigenvector *M* is first extracted using the DML, then the representation matrix is trained using the representation generation module, and lastly, the distance estimation module is used to estimate the distance between the representation vector and the representation matrix to achieve probability-based classification. In the regression branch, the traditional R-CNN method is used for bounding box regression.

**Figure 5:**
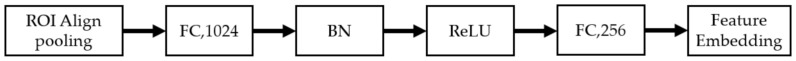
Structure of DML encoding module.

As shown in Figure 5, the eigenvector *M* is input into the DML layer after ROI Align pooling. It enters first into the fully connected layer with 1024 dimensions, and then it is sent into the BN [37] layer and ReLU activation layer [38] for standardization. Lastly, by calculating the embedded eigenvector E through a fully connected layer with 256 dimensions, it is used for subsequent estimation of the sample distance.

The representation generation module is composed of a layer of fully connected layers. The function is to generate a different representation matrix for different categories of detection through training. Specifically, this fully connected layer includes N∗K∗e units, where *N* is the number of categories, *K* is the number of representation vectors of each category, and *e* is the length of every eigenvector. The representation generation module outputs the representation matrix *R*, and $R_{ij}$ is the *j*th representation vector of the *i*th category of samples.

The distance estimation module calculates the distance between the embedded eigenvector *E* and $R_{ij}$ in the representation matrix and determines the category probability according to the distance. The category probability of the foreground object is then calculated, and the distance from *E* to $R_{ij}$ is $d_{ij}\left(E\right)$, $p_{ij}\left(E\right)$ is the probability that the input image belongs to the representation *j* in category *i*, as shown in Equation (3):

(3) $$p_{ij}\left(E\right)\;\propto exp(-\frac{d_{ij}^{2}\left(E\right)}{2\sigma^{2}}).$$

The probability of the image belonging to category *i* can be calculated by the distance matrix, as shown in Equation (4):

(4) $$P\left(C=i|X\right)\;=P\left(C=i|E\right)=\underset{j=1,\ldots ,K}{max}p_{ij}\left(E\right).$$

The above conditional probability is the upper limit of the actual posterior probability and is the maximum value of all combined representations. The background probability is estimated using the lower bound of foreground probability:

(5) $$P\left(B|X\right)\;=P\left(B|E\right)=1-arg\underset{ij}{max}p_{ij}\left(E\right),$$

where $P\left(B|X\right)$ is determined by the maximum foreground probability in all modes.

The regression branch uses the Smooth L1 loss function to measure the distance between the actual box and the predicted box. Loss functions for the classification branch include classification loss and metric loss. The classification loss in this study is the common cross-entropy loss, while the metric loss is used to separate positive and negative samples, and the mathematical model is as shown in Equation (6):

(6) $$L\left(E,R\right)\;=|\underset{j}{min}\;d_{i*j}\left(E\right)-\underset{j,i\neq i^{*}}{min}\;d_{ij}\left(E\right)+\alpha {|}_{+}$$

where $L\left(E,R\right)$ is the loss of the embedded eigenvector extracted by DML, ${\|}_{+}$ is the ReLU function, and $i^{*}$ is the serial number of the accurate sample category. Moreover, $\underset{j,i\neq i^{*}}{min}\;d_{ij}\left(E\right)$ represents the category with the maximal difference from the same category instead of the category with the minimal difference. Equation (6) is the nearest distance between the embedded eigenvector *E* and the accurate category, which is smaller than the nearest distance with the wrong category by a value of *α*, so the iterative learning continues because this condition is not satisfied.

## 4. Detection Method of Key Objects on the Water Surface

In an actual marine environment, there are static objects, such as reefs, ports, and lighthouses, as well as moving objects, such as sailing ships, steamers, and speedboats. Therefore, USVs not only have to detect the category and location information of these objects but also need to perceive the position information of moving objects to provide more accurate information for autonomous navigation and independent operation of other USVs. To solve this problem, we propose a 3D detection method of key objects on the water surface based on multimodal sensor systems for USVs. The detection results are first screened on the basis of few-shot surface object detection. Ship objects are then taken as the target objects for further detection. The position information is obtained and represented by 3D bounding boxes.

**Figure 6:**
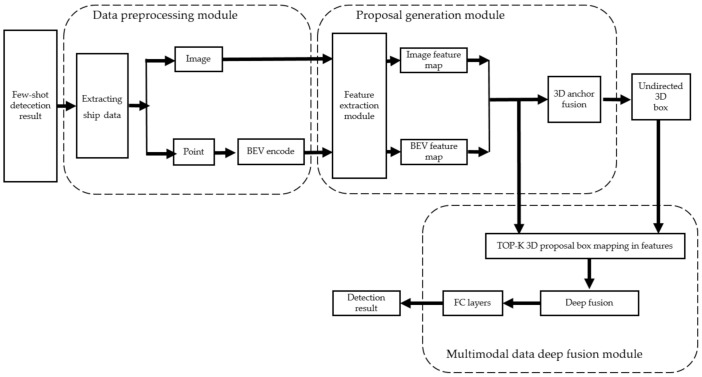
Network structure of water surface key target detection method.

The proposed detection method of key objects on the water surface is a two-stage detection network composed of a region proposal generation module and a multimodal data-deep fusion module, as shown in Figure 6. First, the image data and point cloud data are pre-processed. Second, the pre-processed image data and point cloud data are input into the region proposal generation module to extract eigenvectors and generate 3D region proposal candidate box. Lastly, the eigenvectors of multi-source data are integrated to screen the 3D region proposal candidate box through the classification branch and perform regression of the 3D predicted bounding box.

### 4.1. Data Preprocessing Module

The data pre-processing module processes image data and LiDAR point cloud data. First, the ship data are filtered from the labeled results of few-shot surface object detection. Then, the point cloud data are processed to obtain the BEV. Specifically, the BEV mapping of MV3D is improved by encoding the point cloud data into a six-channel BEV which has a resolution of 700 × 800 and is applicable to USVs’ operation conditions, and the horizontal position information of the 3D point cloud is converted into 2D pixel coordinate information in the BEV. In addition, the solution to compress 3D data into 2D is encoding the height and density information into BEV six-channel information. Specifically, points within $x\in \left[-40,\;40\right]$, $y\in \left[0,\;70\right]$ are selected and divided into grids with a resolution of 0.1 m. Then, points within $z\in \left[0,\;2.5\right]$ are divided into five sections, and 700 × 800 × 5 voxel grids are obtained. Lastly, the heights of these voxel grids are taken as five height channel values of the BEV, and the density of the entire point cloud map is taken as the value of the sixth channel. In detail, we take the value of the highest point in each voxel as the height of whole grid.

### 4.2. Proposal Generation Module

The region proposal generation model first extracts a feature of the input image data and BEV of the point cloud data. Similar to the few-shot surface object detection network, ResNet50 is used as the feature extraction unit. Second, 1 × 1 convolution is performed on the two high-resolution feature maps and are fused using 3D anchors. Lastly, an undirected 3D candidate box is obtained through the NMS layer. The 3D anchors are expressed by six parameters, namely, $\left(cx,\;cy,\;cz\right)$ for the center point, and $\left(dx,\;dy,\;dz\right)$ for the dimensions in the three directions. First, we obtain the 3D anchors through the point cloud BEV, and each group of 3D anchors is sampled at an interval of 0.5 m. Then, the 3D anchors are projected on the image and point cloud BEV to obtain the ROI. After cropping and scaling the two ROIs, the feature maps of the same size are obtained, whose features were integrated at the pixel level using the average addition method. Cross-entropy loss is used as the loss function of label classification in the region proposal generation module, and Smooth L1 is used as the loss function of bounding box regression (of the center point and length, width, and height). The foreground object candidate box is extracted by determining whether the IoU between the 3D anchors and the ground truth is greater than 0.7. Lastly, an undirected candidate box of the top k objects is obtained through the fully connected layer and NMS. Moreover, we used k = 1024, which is an empirical value recommended by Ku [32].

### 4.3. Multimodal Data-Deep Fusion Module

**Figure 7:**
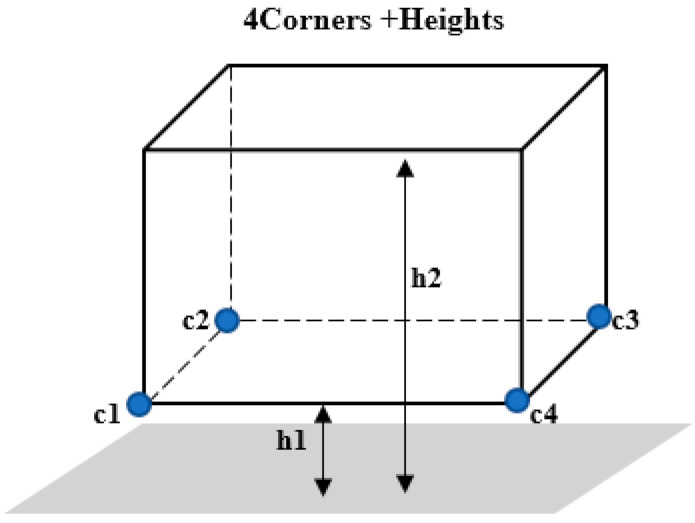
Three-dimensional bounding box encoding.

The multimodal data deep fusion module integrates the 3D candidate box from the region proposal generation module with the image feature map and cloud point BEV feature map, and it predicts the directed 3D bounding box and category using different detectors. The 3D bounding box is encoded by four corners and two height differences, as shown in Figure 7. Unlike the preliminary fusion using 3D anchor mapping, the multimodal data-deep fusion module obtains the 2D candidate box by mapping the top-k 3D candidate boxes onto the BEV feature map and the image feature map. The two 2D candidate boxes are cropped and scaled to achieve the fusion of BEV features and image features using the bit-wise averaging method. Lastly, direction estimation, bounding regression, and label classification are carried out in the fully connected layer, enabling us to obtain directed 3D bounding boxes. The direction estimation constrains the direction of the bounding box using $\left(x_{\theta },\;y_{\theta }\right)=\left(\cos\left(\theta \right),\;\sin\left(\theta \right)\right)$, which eliminates possible ambiguity in the two opposite directions for small boats in the traditional long-edge dependent estimation method.

## 5. Experiments

The experiments were composed of two parts. Since the self-built dataset is small scale, we cannot apply the baseline method on it. Thus, we chose suitable dataset for different experimental purposes of each part. In the first part, the performance of few-shot object detection based on metric learning was evaluated, and ablation experiments were conducted to prove the functions of the modules. Since few-shot detection network takes only image as input, we used ImageNet [39] as our model training set which has always been the benchmark to evaluate the performance of image classification algorithms. In addition, the MS COCO [40] dataset contains a large number of objects, typically with small sizes and complex backgrounds, so it is difficult to carry out visual object detection on the MS COCO dataset. To verify the effectiveness of the proposed method, the MS COCO dataset was chosen to train our backbone network. In the second part, the detection accuracy of key objects on the water surface was evaluated, and the results were compared with other 3D object detection methods. In terms of a test dataset, we chose the KITTI [41], which is obtained from multimodal system and is the largest testing dataset of computer vision algorithms for autonomous driving. Additionally, we chose ships as targets in our experiments and applied the proposed network to our self-built dataset of maritime ships to show its performance on water surface. All of the experiments were performed on a desktop computer with the Ubuntu operating system. The CPU was Intel(R)Core(TM)i9-7920x, and the GPU was TiTanX. Training and testing were carried out using Python on the PyTorch framework.

### 5.1. Few-Shot Object Detection Experiment

First, the weight parameters of the DML and the representation generation module were randomly initialized. Second, end-to-end training of the backbone network was carried out on the MS COCO dataset. Lastly, the detection boxes were ranked according to scores calculated using soft non-maximum suppression (SoftNMS), and the box with the highest score was retained. Each batch of samples was selected randomly. Regularization was performed on the DML output using the L2 paradigm, the number of modes of the representation generation module was set to K = 5, and the exponential function variance $\sigma^{2}=0.5$ of the category probability was calculated.

The few-shot learning module was trained and tested using the 50-way n-shot method. First, each episode trained n random samples of each of the 50 categories and 10*50 random query images on the ImageNet to detect and classify these samples accurately for the few-shot detection task. Second, to maintain consistency, all of the n-shot experiments had 500 random episodes for each $n\in \left\{1,\;5,\;10\right\}$. Each random training episode replaced the category sets to be tested as new categories by replacing the output representation vector of DML with the ROI embedded vector of the extracted image. Lastly, the samples that were never used before were selected from the 50 categories as the query set for the purpose of evaluation.

The few-shot detection experiment was designed based on the R-CNN two-stage detection framework; ResNet50 was used as the backbone, and RepMet was used as the baseline. In the experiment, the backbone network was first trained on the MS COCO dataset, and then 50 random categories of the ImageNet dataset were used for 1-shot, 5-shot and 10-shot training. Lastly, a batch of untrained samples was selected as a query set for testing. The number of testing samples in each category was 500, and the experimental results are shown in Table 2. Average precision (AP) indicates the average of the accuracy corresponding to different recall rates. The mean average precision (mAP) represents the average of the average precision of all of the categories in the dataset, that is, the weighted average of the AP.

The results show that the proposed method achieved better results than LSTD [42] and RepMet [43] in the 5-shot and 10-shot tasks with a large number of samples and the 1-shot task with small number of samples. The reason is that the support set information was introduced to RPN using the attention mechanism, which reduced the interference of the background candidate box and the non-support set candidate box. The classification ability of the model was strengthened by the application of metric learning for the classification task.

Ablation experiments were carried out to show the influence of DML and metric learning on the detection performance of the model on few-shot data. The proposed model was compared with three baseline models: baseline-FT, baseline-DML, and baseline-DML external. Baseline-FT performed fine-tuning on the few-shot dataset using FPN + DCN [29]. Baseline-DML used the fully connected layer of the traditional FPN + DCN, instead of DML as the coding module, to verify the effectiveness of metric learning. The baseline-DML-external model trained the metric learning classifier and DML coding module separately to show whether the end-to-end training is necessary. All of the baseline models were trained and tested on the same dataset, with the same number of episodes.

Thirty categories in the ImageNet (mainly surface objects and vessels) were used in the ablation experiment. The remaining 73 untrained categories of surface objects and vessels were used for testing. For baseline models with the embedded coding module, the category dataset to be tested in each episode was replaced with the new category by replacing the output representation vector of the representation generation module with the ROI embedded vector of the extracted image. The results of the region proposal network were filtered using an IoU threshold of 0.7. For each sample, 2000 ROIs were chosen for the training of the DML coding module. Each episode of the baseline models had five categories of samples, and each category had as many as 50 samples. A total of 500 episodes were carried out for each model, and the mean value of the detection results of 5-way n-shot was calculated. The results are shown in Table 3.

In Table 3, “unseen” refers to the categories that were not used during training, and “seen” refers to the categories that were used in the training. Columns 3 and 4 compare the results of episode-based and non-episode-based training. Based on the results of baseline-DML and baseline-FT, separating the DML coding module from the backbone network resulted in poorer training performance than the baseline models, indicating that a large amount of background information was classified as foreground labels. Thus, the false alarm rate was higher than that of the baseline methods.

### 5.2. Three-Dimensional Detection Experiment of Key Objects on the Water Surface

The detection of key objects on water surface was carried out on the KITTI dataset. The training dataset of KITTI has 7481 pictures, and the testing set has 7518 pictures. The proposed method was evaluated in three difficulty levels, easy, moderate, and difficult, according to the object occlusion rate.

The 3D detection of key objects on the water surface was detected on KITTI using the IoU threshold of 0.7. The results of the 3D object detection accuracy and the average orientation similarity are shown in Table 4. Average orientation similarity (AOS) represents the degree of similarity between the predicted bounding box orientation and the true orientation using the AOS evaluation algorithm.

**Figure 8:**
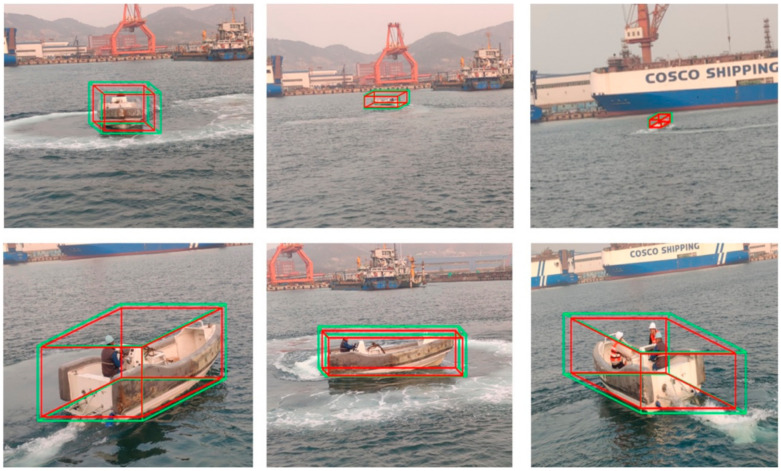
Test result of the object detection tasks in actual application (in different positions, sizes and directions).

The results show that the proposed detection method was superior to the traditional MV3D [11] method in various difficulty levels, and the average orientation similarity obtained with the orientation estimation constraint was also superior to that of the MV3D method. We applied the network to our own dataset of maritime ships, as shown in Figure 8, and our method could obtain accurate three-dimensional position, size and direction of target ship.

## 6. Conclusions

Traditional object detection methods rely heavily on a large amount of labeled data; however, there is a lack of surface object data in complex marine conditions. In this paper, a few-shot surface object detection method was proposed based on the multimodal sensor system for USVs. The traditional object detection method was fused with few-shot learning. Specifically, the background suppression regularization method was introduced in the feature extraction module to extract features of the input image. Then, the object candidate box was generated using the enhanced region proposal network with depth-wise separable convolution. Lastly, the candidate box was screened using the attention mechanism-based coding module, and the few-shot method based on metric learning was used to achieve label classification and bounding box regression and the prediction result. A 3D detection method of key objects on the water surface was proposed based on the few-shot detection method. A directed 3D bounding box of key objects was obtained by multimodal sensor data fusion. Further, comparative experiments were carried out on public datasets to verify the performance of the proposed method. The experimental results indicate that the improved the few-shot object detection model with attention mechanism has better classification ability and a lower false alarm rate than traditional models, which effectively solves the problem that there is no large-scale fully labeled dataset in the task of water surface object detection. The proposed approach achieves a relatively better performance in three sampled sets of well-known datasets, i.e., 2%, 10%, 5% on AP and 28%, 24%, 24% on AOS. Therefore, this study can be potentially used for various applications where the number of labeled data is not enough to acquire a compromising result.

In the future, we plan to optimize the multimodal data fusion method by using multi-angle error weights and assigning weights to error of parameters to obtain more accurate mapping points.

## Figures and Tables

**Table 1:** Advantages and limitations of the main few-shot object detection methods.

Methods	Advantages	Limitations
detection based on fine tuning	High detection accuracy	Susceptible to overfitting on small-scale target domain datasets
detection based on the model structure	Small size, highly accurate candidates	Poor recall rate and reusability
detection based on metric learning	Easy to realize incremental learning	Limited positioning accuracy

**Table 2:** mAP results of 50-way few-shot detection on 50 ImageNet categories.

Model	1-shot (%)	5-shot (%)	10-shot (%)
LSTD [42]	19.2	37.4	44.3
RepMet [43]	24.1	39.6	49.2
The proposed model	24.7	41.2	51.1

**Table 3:** Comparison of mAP results of 5-way few-shot detection with the baseline methods.

Dataset	Method	No Episode Fine-Tuning (%)			With Episode Fine-Tuning (%)		
		1-shot	5-shot	10-shot	1-shot	5-shot	10-shot
**ImageNet (73 unseen)**	Baseline-FT [29]	—	—	—	35	21	59.7
	Baseline-DML [29]	41.3	58.2	61.6	41.3	59.7	66.5
	Baseline-DML-external [29]	19	30.2	30.4	32.1	37.2	38.1
	RepMet [43]	56.9	68.8	71.5	59.2	73.9	79.2
	Ours	57.6	69.5	73.2	59.7	75.1	80.6
**ImageNet (30 seen)**	Ours-trained representatives	—	85.3	—	—	—	—
	Ours-episode representatives	65.5	79.6	82.1	—	—	—

**Table 4:** Test results on samples with different difficulty levels.

Method	Easy		Moderate		Difficult	
	AP (%)	AOS (%)	AP (%)	AOS (%)	AP (%)	AOS (%)
MV3D [11]	79.57	51.69	65.87	44.11	57.83	39.43
Ours	81.23	79.89	75.36	68.17	62.35	65.83

## Data Availability

ImageNet, MS COCO, KITTI are available in the public domain. Our own dataset of maritime ships cannot be shared at this time as the data also forms part of an ongoing study.
